# Exosomes as miRNA Carriers: Formation–Function–Future

**DOI:** 10.3390/ijms17122028

**Published:** 2016-12-02

**Authors:** Xiaojie Yu, Margarete Odenthal, Jochen W. U. Fries

**Affiliations:** 1 Institute for Pathology, University Hospital of Cologne, 50924 Cologne, Germany; xiaojie.yu@uk-koeln.de (X.Y.); jochen.fries@uni-koeln.de (J.W.U.F.); 2 Center for Molecular Medicine Cologne, University of Cologne, 50924 Cologne, Germany

**Keywords:** extracellular vesicle, exosome, cell metabolism, miRNA

## Abstract

Exosomes, which are one of the smallest extracellular vesicles released from cells, have been shown to carry different nucleic acids, including microRNAs (miRNAs). miRNAs significantly regulate cell growth and metabolism by posttranscriptional inhibition of gene expression. The rapidly changing understanding of exosomes’ formation and function in delivering miRNAs from cell to cell has prompted us to review current knowledge in exosomal miRNA secretion mechanisms as well as possible therapeutic applications for personalized medicine.

## 1. Introduction

Our understanding of cell-to-cell communication has been revolutionized by the observation that extracellular vesicles (EVs) derived from cells are not only capable of transferring miRNAs, mRNAs, and proteins in a paracrine (between cells of the same origin) manner, but also in an endocrine (to distant target cells) manner. EVs have been characterized by size into three different classes (exosomes: <100 nm; microvesicles: <1000 nm; apoptotic bodies: 1–4 µm) [1]. Many studies have shown that EV uptake is mediated by clathrin- and caveolin-dependent endocytosis, macropinocytosis, or lipid raft–mediated endocytosis [2,3]. Previously, however, the term “microvesicle” was used to define the conglomerate of these three different vesicles. Their isolation was based on ultracentrifugation, modified for their biophysical properties, followed by electron microscopic analysis [4]. This, however, could lead to ambiguous experimental outcomes by using microvesicles to analyze exosome functions. Currently, advanced techniques utilize ultrafiltration followed by affinity purification [5]. Exosomes, in particular, can be affinity-purified using tetraspanins as the surface antigen, while microvesicles can be identified by their surface expression of phosphatidylserine [6]. In this review we will focus on the most recent advances in our understanding of exosome function, with particular emphasis on liver disease and the potential relevance for personalized medicine. Important aspects will be highlighted as a take-home message (THM) at the end of each paragraph.

THM: Extracellular vesicles transport miRNA in a paracrine and endocrine manner. Questions regarding the cellular options of producing either microvesicles or exosomes and their difference in miRNA composition and number are still unknown.

Exosomes were discovered during mammalian reticulocyte maturation, shedding from the plasma membrane [7]. They are membrane vesicles with diameters ranging from 40 to 100 nm which are formed in the endocytic compartment (“multivesicular body”, MVB) during endosome maturation by inward budding [8]. Increasingly more cell types have been found to secret exosomes, including hepatocytes, dendritic cells, red blood cells, mast cells, epithelial cells and tumor cells. Exosomes bear intraluminal and transmembrane proteins [9], such as heat shock proteins (HSP70, HSP90), tetraspanin proteins (CD9, CD63, CD81), ceramids, and cholesterol [10]. Because of the exosome surface proteins, they are suggested to interact with recipient cells in a ligand-receptor manner via endocytosis and thus deliver incorporated materials from cell to cell [11]. It is generally believed that exosome release is due to the fusion of the MVB and plasma membrane, followed by intraluminal vesicle release [12]. Microarray assessment of exosome RNA shows that around 1300 different RNAs are enclosed in exosomes released by human mast cells. These exosomes shuttle RNA, including miRNAs, which are delivered into recipient cells, some of which have even been proven to be functional [13].

THM: Exosomes interact with the cell via their surface proteins in a ligand-receptor manner. Tetraspanins such as CD63 may be used for affinity purification.

## 2. MicroRNA Loading into Exosomes

**Figure 1:**
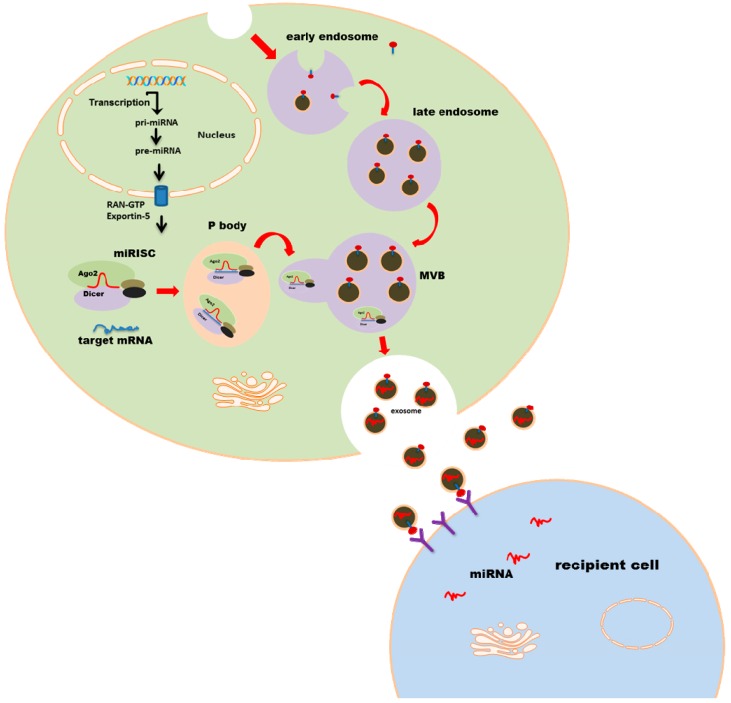
Mechanism of exosomal miRNA secretion. Pri-miRNA is initially transcribed from the genome, processed by Drosha to pre-miRNA, and is then transported into cytoplasm via Exportin-5 where mature miRNA is formed. Mature miRNA is integrated into the miRISC complex and therefore targets mRNA. The P-body fuses with late endosomes and releases miRNA-containing exosomes. Exosomes are further taken up by the receptor-ligand interaction, regulating a series of gene expressions in recipient cells.

MicroRNAs (miRNAs) are small, endogenous, non-coding nucleotides, approximately 22 nt long, regulating gene expression post-transcriptionally. Primary miRNAs (pri-miRNAs) are transcribed by RNA polymerase II. Nuclear RNase Drosha processes the pri-miRNAs into a hairpin precursor miRNA (pre-miRNA). Pre-miRNAs are transported into the cytoplasm, where the hairpin is cleaved off, resulting in double-stranded mature miRNA [14]. One strand is integrated into Argonaute (Ago) protein–containing miRNA-induced silencing complex (miRISC) and interacts with target miRNA transcripts, which leads to the repression of gene expression [15] (Figure 1).

Since the Agonaute proteins are crucial for miRNA function, they have also been considered as a fundamental carrier of miRNA and may also be involved in exosomal loading and miRNA release. However, recent studies have shown that exosomal miRNAs are free of Ago2, and more than 90% of Ago protein–bounded miRNAs are independent of extracellular vesicles [16,17]. This is consistent with the discovery that exosomal miRNA is completely independent of miRISC. Instead, they are specifically recognized by individual proteins, such as hnRNPA2B1 and hnRNPA1 which recognize the specific binding motif of miRNAs and selectively load them into exosomes [18]. Furthermore, mature miRNA, GW182 and Ago proteins form the GW body which captures untranslated target mRNA and forms a specific cytoplasmic foci (the so-called P-body) [19,20], followed by its fusion into MVBs. However, Gibbings et al. found neither Ago proteins nor target mRNAs in exosomes [21]. The processing bodies, the so-called P-bodies, are cytoplasmatic foci, complexing many enzymes involved in RNA turn-over. The dual immunofluorescence assay shows that Ago interacts with the Dcp1a and Dcp2 proteins of the P-bodies and, therefore, localizes miRISC to the P-bodies [22]. Thus, it is very likely that miRISC is initially directed to the P-bodies after miRISC binds with its target mRNA. This hypothesis is further reinforced by the discovery that miR-122’s target, cationic amino acid transporter (CAT-1), can be relieved from miR-122–induced inhibition under stress conditions. Furthermore, this depression is accompanied by its release from the P-bodies [23]. However, the inward budding of endosomes and vesicle sorting require sequential assembly of the endosomal sorting complex needed for transport (ESCRT). Either blocking of endosome formation or knock-down of ESCRT genes impairs microRNA activity [21], which indicates that miRNA-induced gene repression requires, at least in part, ESCRT sorting and MVB functionality. This is in agreement with the finding that active miRISCs are physically and functionally incorporated into early endosomes [24]. It is reasonable to accept that some ‘unwanted’ miRNAs are captured by the miRISC complex and transported via ESCRT to early endosomes, where miRNAs are further redirected to intraluminal vesicles (ILV) (Figure 1). However, it still remains to be answered how miRNA disintegrates from miRISC and is selectively incorporated into late endosomes (namely MVBs) before exosome release.

THM: miRNA composition/loading into exosomes mainly depends on the surface molecules of exosome membranes, the endosomal sorting complex required for transport (ESCRT), and the specific binding motifs of the miRNAs themselves.

## 3. Exosome-Mediated Regulatory Effects in Liver

Exosomes play an essential role in maintaining cell homeostasis during liver damage. It is generally acknowledged that circulating miR-122 is released from hepatocytes regardless of the type of liver injury [25,26,27,28]. It has also been proposed that liver cells release exosomes, containing miRNAs, as a defense mechanism [29,30,31]. Various studies have shown that miR-122 is released after chronic and acute liver damage [32,33,34]. However, due to different stimulants, miR-122 is released under different formations. Alcohol-induced liver injury induces a significantly higher amount of exosomal miR-122 release [35]. In parallel, the intracellular miR-122 level is strongly downregulated [36]. Furthermore, alcohol-induced exosome release is involved with caspase-3 activation and it contains the macrophage-activating CD40 ligand [37]. As well, in viral liver hepatitis, miR-122 is secreted together with the hepatitis viron and is transferable to non-infected cells [38]. However, during drug-induced liver injury (LIDI) circulating miR-122 binds with proteins and is highly independent of exosome sequestration [25]. Because circulating miR-122 is independent of the type of liver injury [25,26,27,28], it is conceivable that the type and degree of liver injury determine exosomal miRNA secretion as part of maintaining liver homeostasis. Hepatocyte-secreted exosomes contribute to cell proliferation, liver repair and liver regeneration [39]. Furthermore, liver disease progression also contributes to miRNA release. Hepatocellular carcinoma (HCC) patients release lower amounts of exosomal miR-122 compared with controls [40]. More importantly, exosomal miR-122 has been shown to be internalized by HCC cells and decrease their miR-122 expression level [41].

Hepatocytes not only secret exosomes for cell survival, but they are also regulated by exosomes from different sources. Currently, exosomes from mesenchymal stem cells (MSCs) have attracted great attention worldwide and have been proposed as one of the most efficient liver cancer therapy options [42,43]. Research discoveries have shown that exosomes from MSCs exhibit potent protective activities towards liver diseases as observed in: promoting liver regeneration [44,45], suppressing the liver inflammatory response [46], alleviating liver fibrosis [44,47], increasing the sensitivity of liver cancer cells to chemotherapeutic agents [48], and promoting the anti-tumor activities of MSCs [49]. Besides, MSC-secreting exosomes, carrying miRNAs, reduce the growth [50] and suppress angiogenesis [51] of breast cancer cells. For non-liver cells, MSC exosomes have been demonstrated to inhibit macrophage activation [52], promote skeletal muscle regeneration [53] and increase cell proliferation of renal tubular epithelial cells [54]. These proofs altogether lead to the conclusion that MSCs release exosomes to regulate cell metabolism, such as to inhibit tumor growth and sustain normal tissue function. This notion is further reinforced by the discoveries that exosomes released from stem cells modulate the cell phenotype [55,56,57] and contribute substantially to tumor metastasis [58,59]. Therefore, further studies will be needed to determine the exosome secretion pattern released by cancer patients, as a clinical prognosis of tumor metastasis, and an effective criterion for taking preventive measures.

THM: The type and degree of (liver) injury determine exosomal miRNA secretion as part of maintaining homeostasis. Released liver miRNAs may modify the reaction pattern of other cells such as hematopoietic or endothelial cells. Progression of chronic diseases such as hepatitis and liver fibrosis will change secretion patterns of miRNAs, exosomal loads, and may lead to changes in therapeutic responses.

## 4. Exosomal miRNA Uptake

Recent studies indicate that exosome uptake might not be a random process; rather, it is mediated by endocytosis, micropinocytosis or phagocytosis, which have been extensively described before [60,61]. Among them, heparan sulfate proteoglycans (HSPG) [62] and caveolae-mediated uptake associated with cholesterol-enriched membrane microdomains have been described in glioblastoma cell lines [63]. HSPG on the surface membrane of cancer cells has been proven as an exosome fusion receptor; however, inhibiting HSPG synthesis in cancer cells only partially decreases exosome uptake [62]. Therefore, exosome fusion with the membrane depends on intact HSPG synthesis, suggesting that it is a coordinated process in which additional HSPG-independent mechanisms of internalization might be involved.

Tetraspanins [64] were reported as highly abundant components in exosomes from different tumor entities such as lymphoma [65,66], colon carcinoma [67], melanoma [68], gastric adenocarcinoma [69], lung adenocarcinoma [70,71], and gastrointestinal stromal tumor [72]. CD63 is suggested as a surface marker [65,66] and it locates mainly in MVBs and lysosomes. However, plasma membrane CD63 protein has been found to be negatively involved in the progression of metastatic diseases [67]. Considering that exosome biogenesis starts from the inward budding of plasma membrane, it looks tantalizingly believable that exosome secretion associates with cell proliferation and metastasis. Furthermore, CD63 has been shown to interact with tissue inhibitor of metalloproteinase 1 (TIMP1) [68], which interacts with transmembrane protein integrin β and subsequently activates the PI3K/Akt pathway [73]. That is in accordance with the discovery that exsomes, which are derived from gastric cancer cells, activate the PI3K/Akt pathway, leading to the promotion of cell proliferation [69]. Besides, CD63 also binds with the kit receptor tyrosine kinase [74], which has been found to be enclosed in gastrointestinal stromal cell–derived exosomes and associates with tumor cell invasion [72]. That is generally inconsistent with the report that mast cell–derived exosomes transport tyrosin kinase to lung cancer cells and promote cell growth [70]. These pieces of evidence indicate that CD63 contributes not only receptor-ligand–mediated exosomal uptake but also scrutinizes exosome sequestration, which ultimately determines exosome functionality. Indeed, overexpression of TIMP1 in lung adenocarcinoma cells induces miR-210 accumulation in exosomes [71].

Integrin exists ubiquitously on cell membranes, serving as bridges for extracellular matrix (ECM) and signal transduction to the cells [75]. For focal adhesion, integrin and heparan sulfate (HS) proteoglycans (HSPG) cooperate to connect cytoskeleton and ECM [76]. Recently, exosomes from adipocytes integrated into different liver cells [77] were described as causing the upregulated expression of integrin αvß5 and matrix metalloproteinase. Considering the unsolved puzzle of organotropic metastases, it seems particularly noteworthy to mention the work of Hoshino et al. [59]. They have shown that tropism for liver, lung, or brain metastases is based on the uptake of tumor-derived exosomes into the respective normal resident cells. This uptake is mediated by a distinct integrin expression pattern on the exosomal surface, directing the exosomal uptake to the liver (αvβ-5 integrin) or the lung (α6β4/α6β1 integrins) (Figure 1). Finally, the process of organotropic metastases may be further supported by creating a receptive niche through exosomes, which prime naïve cells in the specific organ itself [58]; for instance, HCC tumor cell exosomes influence the neighboring cells through transfer of oncogenic proteins and RNAs [58]. Niche formation has also been described as an effective metastatic mechanism for pancreatic cancer exosomes [58].

THM: Exosomes contain miRNA profiles which may allow the determination of the status of the cells and their origin. Its surface molecules may determine the specificity of uptake in the target organ and explain metastatic organotropism.

## 5. Regulatory Functions of Secreted miRNAs in Cancer

Because exosome proteins control the vesicle contents, in order to maintain their oncogenesis and metastasis, cancer cells tend to sequester antitumor miRNAs in vesicles and load them to exosomes. miR-146 has been proved as a tumor suppressor in prostate, glioma, breast and pancreatic cancers [78,79,80,81]. Indeed, overexpression of tumor suppressor miR-146 in stromal cells triggers its exosomal release [82].

In addition, the tumor suppressor miRNA let-7 is highly concentrated in the exosomal fraction from highly metastatic gastric cancer cells in comparison with that from less metastatic gastric cancer cells [83]. Intravenous exosome injection can deliver let-7 to breast tumors in vivo [41]. It signifies a possible link between the loss of tumor suppressor miRNA in cancer and exosome secretion. However, this regulation mechanism is not solely present in cancer cells. Thus, it has also been described during TGF-β–induced migration of keratinocytes, whose miR-198 expression level was downregulated in response to TGF-β stimulation [84]. Besides, miR-198 has been shown as a potent tumor suppressor in lung, liver and colorectal cancer [85,86,87,88], and it also has been shown to be secreted by T-lymphoblastic cells via exosomes [18].

Besides carrying tumor suppressor miRNAs, exosome function is also involved in the intercellular transmission of oncogenically acting miRNAs. For example, in many cancer cells, oncogenic miR-21 [89,90] expression is upregulated and secreted via exosomes, promoting tumor progression and metastasis [91,92]. However, it is generally acknowledged that exosomes do not include only one kind of miRNA; rather, a wide spectrum of miRNAs is simultaneously transported via exosomes. Therefore, the exosomes’ inducing effect depends largely on the mixture of miRNAs [13]. Exosomes containing oncogenic miR-21 and tumor suppressor miR-29a bind toll-like receptors (TLR) in immune cells, leading to activation of the TLR-mediated NF-κB pathway and the secretion of prometastatic inflammatory cytokines which ultimately contribute to tumor growth and metastasis [93]. However, loading various miRNA species has not been completely understood yet and further mechanistic insights would rely on technologies that differentiate miRNA-specific exosomes from a mixture of exosomes.

THM: Tumor suppressor miRNAs are more preferentially sequestered by vesicles and secreted as exosomal miRNAs, not only inhibiting the growth of cells in the surrounding environment but also targeting cells at a long distance. The miRNA profiles of exosomes may enable us to analyze their targeted cell groups and possible functions.

## 6. Outlook: miRNAs Secreted in Exosomes as Diagnostic Tools

Currently, the diagnostic potential of the analysis of circulating exosomes and their microRNAs has been recognized they are used as biomarkers in a variety of diseases. In particular, in a wide variety of different acute and chronic liver diseases, miRNA and exosomal vesicles are discussed as putative early prognostic indicators [39,40,94,95,96,97,98,99]. Beyond specific diseases, the use of exosomes could be envisioned as a tool to determine the functional reserve capacity of an organ, i.e., its remaining ability with increasing age to perform adequately, particularly under stress such as anesthesia and surgical intervention. Towards this goal, investigators have studied the change in exosomes in the aging process of an organ/organ system such as the brain [100], the eye [101], the salivary glands [102], the kidney [103], and the prostate [104]. The degenerative changes in these organs have in common the fact that we do not possess any meaningful serum markers, which would allow monitoring such organ alterations. Studies which would be of high interest for the future establishment of novel diagnostic tools would include investigations at the exosome/miRNA level of the development and growth of organs as well as follow-ups over an age range and potential ethnological differences in organ metabolism (i.e., liver detoxification). Since the methods of isolation using molecules specific for either exosomes (such as tetraspanin) or microvesicles (such as phosphatidylserine) might finally enable us to highly purify exosomes, several unanswered aspects may be explored. One of these aspects is the ratio between microvesicles and exosomes simultaneously released from the same cell and their changes due to physiological, e.g., stress, and pathological processes, e.g., toxins and carcinogenesis. Furthermore, knowing more about the membrane composition of these vesicles would greatly help in classifying their origin and better understanding the cause of their production. Simultaneously, this would also enhance our understanding of the selection process of miRNA (and other molecules) as content and vice versa.

Finally, if exosomes are able to modify the cancer environment or provide a specific niche for metastases, their membrane and miRNA analyses may be helpful in understanding the as yet unexplainable behavior of cancers with identical morphologic appearances. Here exosomal miRNA-based manipulation of signal pathways in the target cell and modification of oncogenic factors could well be responsible for several mysteries: (i) observed early cancer recurrences in spite of complete resection in the immediate vicinity of the original tumor; (ii) metastases via lymphangiosis and hemangiosis due to endothelial cell manipulations; and (iii) the development of early therapeutic resistance not based on genetic mutations.

THM: Exosomal miRNA opens a new era of molecular diagnostic tools. It has the therapeutic potential to determine functional aspects, such as organ aging, as being the basis for individual patient tolerance of therapeutic intervention. Furthermore, this tool may determine the influence of physiological and pathological processes in exosome formation. To this end, more details about the interaction between formation and function are necessary in order to apply this knowledge for individualized patient management.
